# Reduced ^15^N Losses by Winter and Spring Night-Warming Are Related to Root Distribution of Winter Wheat

**DOI:** 10.3389/fpls.2019.00771

**Published:** 2019-06-19

**Authors:** Chenxi Hu, Jinghong Yu, Shuzhen Sun, Yanyan Yan, Hua Guo, Zhongwei Tian, Dong Jiang, Weixing Cao, Tingbo Dai

**Affiliations:** Key Laboratory of Crop Physiology Ecology and Production Management of Ministry of Agriculture, Nanjing Agricultural University, Nanjing, China

**Keywords:** asymmetric warming, root weight density, nitrogen recovery, nitrogen residual, grain yield

## Abstract

To develop efficient N management strategies for high wheat NUE and minimizing the environmental impact of N losses under asymmetric warming, ^15^N micro-plot experiments were conducted to investigate the effects of night-warming during winter (warming by 1.47–1.56°C from tillering to jointing), spring (warming by 1.68–1.82°C from jointing to booting), and winter + spring (warming by 1.53–1.64°C from tillering to booting) on root growth and distribution of winter wheat, the fates of ^15^N-labeled fertilizer, and their relationships in 2015–2017. The results showed that night-warming increased the recovery of basal ^15^N and top-dressed ^15^N, while reduced the residual and loss of basal ^15^N and top-dressed ^15^N. The losses decreases of top-dressed ^15^N were higher than those of basal ^15^N, indicating that night-warming reduced losses of fertilizer ^15^N mainly by reducing losses of top–dressed ^15^N. Moreover, pre-anthesis root dry matter accumulation rate in 0–60 cm soil layer were promoted, resulted in improved root biomass and root/shoot ratio, which favored increasing recovery of fertilizer ^15^N and reducing losses of fertilizer ^15^N. Furthermore, residual fertilizer ^15^N content in 0–100 cm soil layer was reduced, which was associated with improved root weight density in 0–60 cm soil layer, resulted in reduced leaching losses of fertilizer ^15^N. The path analysis showed that root dry matter distribution in 0–20 cm soil layer was the most important in contributing to reducing losses of total fertilizer ^15^N compared with other soil layers. Two years data showed that winter and spring night-warming gave better root growth and distribution in 0–20 cm soil layer, resulted in reduced the losses of fertilizer ^15^N and improved the recovery of fertilizer ^15^N, while maximizing grain yield of winter wheat, and winter + spring night-warming resulted in higher advantages than winter night-warming and spring night-warming.

## Introduction

The global mean air temperatures have increased in the past 100 years and are projected to increase 0.3–4.8°C by the end of this century (IPCC, 2014). Global air temperature often exhibits seasonal and diurnal warming asymmetry. Air temperature increases are higher in winter and spring than in autumn and summer, and in nighttime than in daytime (Clarke and Zani, 2012; Xia et al., 2014; Andersen et al., 2017; Freixa et al., 2017). Winter wheat is the predominant crop in the world, which is mainly cultivated in winter and spring (Chen et al., 2014). Therefore, this kind of asymmetric warming is expected to profoundly influence wheat production.

Nitrogen fertilizer is the most important to improve grain yield and quality of wheat. Nitrogen fertilizers are applied annually in all countries to increase yield, while the worldwide N use efficiency (NUE) in wheat systems is 30–50% (Raun and Johnson, 1999; Raun et al., 2002). Wheat crops with low NUE result in serious environmental impacts through N losses, such as eutrophication of surface waters, nitrate pollution of groundwater and greenhouse gas emissions (Foulkes et al., 2009; Townsend and Howarth, 2010; Miao and Zhang, 2011; Congreves et al., 2016). Warming was reported to have effects on the growth, development, and physiological processes of plants (Asseng et al., 2013), which exerted a profound impact on wheat NUE (Liu et al., 2013; Zhang et al., 2013). However, most of these studies were focused on daily mean air temperature and entire growth period, and did not take into consideration of warming asymmetry. Thus, it is essential to improve wheat NUE and minimize the environmental impact of N losses in the future asymmetric warming.

Fertilizer N in wheat-soil system had three fates: uptake by wheat, residual in soil, and loss from the wheat-soil system (Shi et al., 2012). Several studies reported that the plant N uptake could be affected by climate warming (Sardans et al., 2008; Kim et al., 2011; Nam et al., 2013), which could potentially affect the residual and loss of N fertilizer. For example, Cheng et al. (2010) reported that high night temperature increased plant above ground biomass, and had no effect on plant N concentration, resulted in significantly improved plant N uptake of rice. Recently, it is reported that plant N uptake of wheat was 17–43% higher in night-warming treatment during the jointing, anthesis, and maturity stages (Zhang et al., 2013). However, most of these studies were focused on the apparent N recovery of plant, and the fates of N fertilizer in the wheat-soil system in response to asymmetric warming are still not clear. For efficient fertilizer N utilization in the future asymmetric warming, use of ^15^N-labeled fertilizer is an effective measure to quantify the fates of N fertilizer (Blankenau et al., 2000).

Root growth and distribution have an important effect on the plant N uptake and N losses in the wheat-soil system, and good root growth and distribution help improve plant N uptake and decrease N losses (Shi et al., 2012; Wang et al., 2014). It is reported that wheat growth, development, and grain yield could directly be influenced by climate warming (Liu et al., 2013; Chen et al., 2014; Cai et al., 2016). As the root growth and distribution are closely related to wheat aboveground growth and development (Qin et al., 2012), it is expected that root growth and distribution will be indirectly affected by climate warming. Furthermore, soil temperature is a major environmental factor influencing plant root growth (Lahti et al., 2005). Root growth and distribution will also be directly affected by climate warming, as air temperature increase will lead to soil temperature increase (Kelly et al., 2011). Several studies have conducted to learn about root growth and distribution in response to climate warming (Dukes et al., 2005; Zhou et al., 2012; Qiao et al., 2015). For example, Xu et al. (2015) reported that warming treatments induced the downward transport of soil moisture, resulted in increased root biomass in deep soil layers. Bai et al. (2010) reported that under ambient precipitation root production and standing root biomass were increased in response to climate warming in the semiarid temperate steppe. Recently, Hou et al. (2018) reported that warming significantly increased root biomass in the 0–30 cm soil layers under two tillage systems, and in till system root biomass was increased in the deeper soil layers (10–20 and 20–30 cm), while in no-till system root biomass was increased in the surface layer (0–10 cm). However, most of these studies were focused on total root morphology and biomass, and the response of root growth and distribution in different soil layer to asymmetric warming remains unknown.

We have reported that winter and spring night-warming improved root extension and soil nitrogen supply, ultimately increasing N uptake and utilization of winter wheat (Hu et al., 2018). The objectives of this study were (1) quantify the fates of ^15^N-labeled fertilizer during different growth periods under asymmetric warming and (2) clarify the responses of root growth and distribution in different soil layers to asymmetric warming and their relationship with the fates of ^15^N-labeled fertilizer. The results are intended to develop efficient N management strategies for high wheat NUE and minimizing the environmental impact of N losses under future warming condition.

## Materials and Methods

### Experimental Design

**FIGURE 1:**
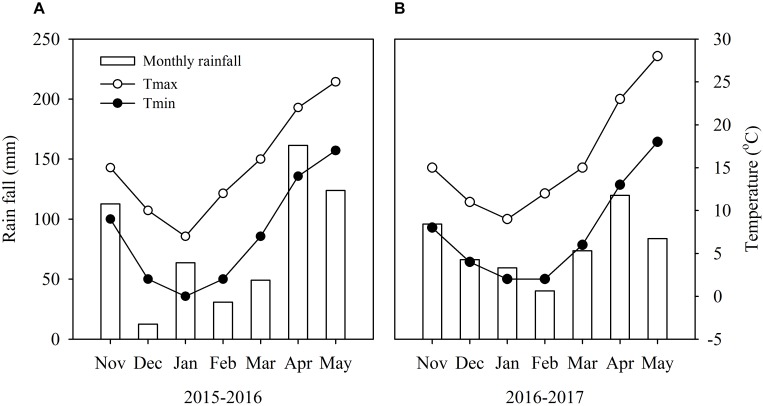
Monthly mean rainfall and mean maximum and minimum temperatures during the wheat growing season from 2015 to 2016 **(A)** and 2016 to 2017 **(B)** in Nanjing, China.

Field experiments were carried out in 2015–2017 in Nanjing (32°04′N, 118°76′E), China, using Yangmai-13 (vernal type) winter wheat cultivar. In this region, the annual mean temperature and rainfall were 15°C and 1000 mm, and the annual mean incoming solar radiation was 4530 MJ m^−2^. This cultivar is one of the most commonly planted cultivars in the Yangtze River Basin. Weather conditions during the experimental period and soil properties of 0–100 cm soil layer before sowing are shown in Figure 1 and Table 1.

**Table 1:** Soil properties of experimental site at the beginning of experiment in two growth seasons.

Growing season	Soil layer (cm)	Soil Texture	Organic matter (g kg^−1^)	Total N (g kg^−1^)	Olsen-P (mg kg^−1^)	Exchangeable K (mg kg^−1^)	Nitrate N (mg kg^−1^)	Ammonium N (mg kg^−1^)	PH
2015–2016	0–20	Loamy clay	10.95	1.33	9.85	74.30	8.54	3.43	7.48
	20–40	Loamy clay	10.45	1.21	8.28	68.39	5.33	2.68	7.55
	40–60	Loamy clay	9.33	1.04	7.57	63.70	2.71	2.33	7.58
	60–100	Loamy clay	9.29	0.87	6.45	60.48	2.38	2.17	7.69
2016–2017	0–20	Loamy clay	11.36	1.28	10.14	72.27	9.38	3.80	7.47
	20–40	Loamy clay	10.16	1.19	8.41	70.13	6.51	3.14	7.72
	40–60	Loamy clay	9.23	0.98	7.46	64.55	3.57	2.62	7.82
	60–100	Loamy clay	9.12	0.91	5.97	59.25	2.92	2.38	7.85

**FIGURE 2:**
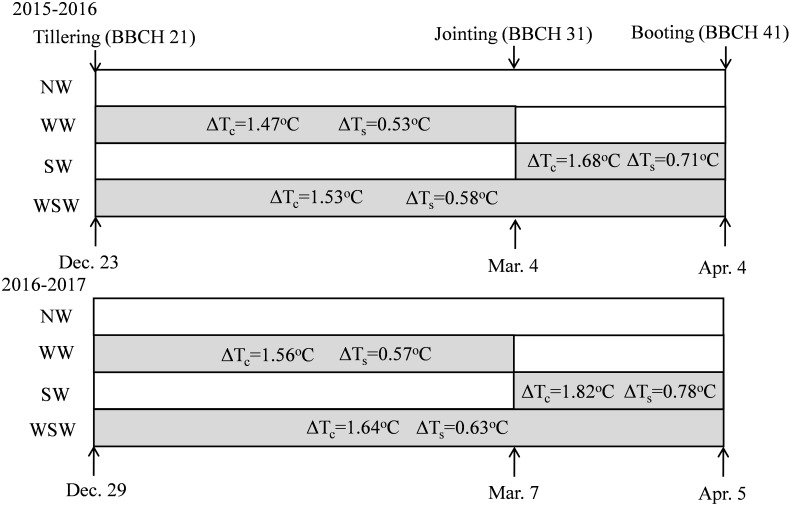
Schematic representation of experimental design and treatments. NW, WW, SW, and WSW refer to no warming control, winter night-warming, spring night-warming, and winter + spring night-warming, respectively. ΔT_c_ and ΔT_s_ refer to the increase in the mean night temperature of canopy and 5 cm soil layer between treatments and the control, respectively. Mean night temperature is the mean in all temperature data on a 10-min interval from 19:00 to 07:00 h.

The experiment included macro-plot experiment and ^15^N micro-plot experiment. The macro-plot experiment was a randomized complete block design and consisted of three replicates. Four warming treatments: winter night-warming treatment (WW), spring night-warming treatment (SW), winter + spring night-warming treatment (WSW), and no warming control (NW). The treatment details were described by our previous study (Hu et al., 2018) and thus are briefly introduced here. The warming treatment was based on the technique of passive night-warming and achieved temperature increases by covering with a plastic membrane from 19:00 h to 07:00 h of the next day. The warming facility was 5 m in length, 3 m in width, and 2 m in height. The experimental design is shown in Figure 2. The plot area was 8 m^2^ (2 m × 4 m), at a seeding rate of 225 plants m^−2^, with a 0.25 m row spacing. In the two wheat growth seasons, 120 kg N ha^−1^,105 kg P_2_O_5_ ha^−1^, and 150 kg K_2_O ha^−1^ were supplied in all plots before sowing (BBCH 00), and another 120 kg N ha^−1^ was supplied equally as a top-dressing at the jointing (BBCH 31) and booting (BBCH 41) stages. N fertilizer was added in the form of urea (46% N). Sowing dates were 4 November in 2015 and 9 November in 2016.

The ^15^N micro-plots were set within macro-plots by polyvinyl chloride (PVC) tubes with 25 cm diameter and 105 cm height to quantify the fates of N fertilizer and monitor the root growth and distribution, this method was also used by our previous studies (Shi et al., 2012; Hu et al., 2018). The ^15^N micro-plot experiment designed same as the macro-plot experiment. To keep the soil conditions in ^15^N micro-plot similar to those in macro-plot, soil was dug out and separated into four layers: 0–20, 20–40, 40–60, and 60–100 cm, and then was backfilled into the PVC tube in the correct order. Next, the PVC tubes were buried into field plots with their top edges 5 cm above the ground. All ^15^N micro-plots were supplied 24 g N m^−2^ with following treatments: (1) 50% basal (^15^N-labeled fertilizer)–50% top-dressing (normal fertilizer), and (2) 50% basal (normal fertilizer)–50% top-dressing (^15^N-labeled fertilizer) with three replicates. In total, there were 60 micro-plots in the field. N fertilizers were used with ^15^N-enriched (10.16 at% excess ^15^N) ammonium sulfate (Shanghai Chemical Industry Institute) and normal ammonium sulfate. Basal fertilizers were applied by mixing with 0–20 cm soil before sowing (BBCH 00). Top-dressed N fertilizers were applied equally by dissolving in 100 ml water at the beginning of jointing (BBCH 31) and booting (BBCH 41) stage. Every micro-plot planted eleven seedlings. To reduce edge effects, around the micro-plots, wheat seedlings were planted.

### Sampling Methods and Analysis

The samples for night-warming treatments were taken in the micro–plots at jointing (BBCH 31), anthesis (BBCH 65), and maturity (BBCH 89). Plant samples were separated into leaves, culm, chaff, and grain (maturity). Root samples were separated into 0–20, 20–40, 40–60, and 60–100 cm soil layers by washing out any soil. All separated samples were oven-dried at 70°C to constant weight to estimate dry matter accumulation. Soil samples were taken at four layers: 0–20, 20–40, 40–60, and 60–100 cm. Each soil sample was separated into two parts. One part was oven-dried at 105°C for determination of water content. The other part was dried under natural conditions for determination of ^15^N enrichment. Plant and soil samples were taken outside the micro-plot (more than 1 m away) for determination of the natural ^15^N enrichment. The plant and soil samples were finely ground to 100 μm and analyzed for total N and ^15^N enrichment by an automated continuous flow Isotope Cube (Elementar, Germany) coupled with a continuous flow mass spectrometer (Isoprime, United Kingdom) using Dumas flash combustion.

### Calculation Methods

(1) $$\mathrm{Root weight density}\;(\mathrm{g m}-3)=\frac{\mathrm{Root weight in soil layer}}{\mathrm{Volume of soil layer}}$$

The percentage of N derived from fertilizer were calculated by the following equation (Malhi et al., 2004):

(2) $$\text{Ndff}(\%)=\frac{\text{c-b}}{\text{a-b}}\times 100$$

where a is the atom% ^15^N in the labeled fertilizer, b is the atom% ^15^N in the plant or soil receiving no ^15^N, and c is the atom% ^15^N in the plant or soil receiving ^15^N.

Plant N uptake and the fates of the N fertilizer were calculated by the following equations:

(3) $$\mathrm{Plant N uptake}\;({\mathrm{g m}}^{-2})=\frac{\mathrm{plant dry matter accumulation}\times \mathrm{plant N concentration}}{100}$$

(4) $$\mathrm{Plant N uptake from fertilizer}\;({\mathrm{g m}}^{-2})=(3)\times \mathrm{Ndffplant}$$

(5) $$\mathrm{Plant N uptake from soil}\;({\mathrm{g m}}^{-2})=(3)-(4)$$

(6) $$\mathrm{Fertilizer N residual}\;({\mathrm{g m}}^{-2})=\mathrm{soil thickness}\;(\mathrm{cm})\times \mathrm{soil bulk density}\;(g\;\mathrm{cm}-3)\times \mathrm{soil N concentration}\times 10\times \mathrm{Ndffsoil}$$

(7) $$\mathrm{Fertilizer N loss}\;({\mathrm{g m}}^{-2})=\mathrm{N fertilizer application amount}-(4)-(6)$$

(8) $$\mathrm{Fertilizer N recovery percentage}\;(\%)=\frac{\left(4\right)}{\mathrm{N fertilizer application amount}}\times 100$$

(9) $$\mathrm{N residual percentage}\;(\%)=\frac{\left(6\right)}{\mathrm{N fertilizer application amount}}\times 100$$

(10) $$\mathrm{N loss percentage}\;(\%)=\frac{\left(7\right)}{\mathrm{N fertilizer application amount}}\times 100$$

### Statistical Analysis

The analysis of variance (ANOVA) was conducted using SPSS software (SPSS 17.0, SPSS, Inc., United States). Means of different treatments were compared by the least significant difference (LSD) at 5% level. Path analysis was performed to assess the relationship between root dry matter distribution in different soil layers and losses of total fertilizer ^15^N. Graphics were drawn by using SigmaPlot software (SigmaPlot 10.0, Systat Software, Inc., United States).

## Results

### Root Biomass and Root/Shoot Ratio

**FIGURE 3:**
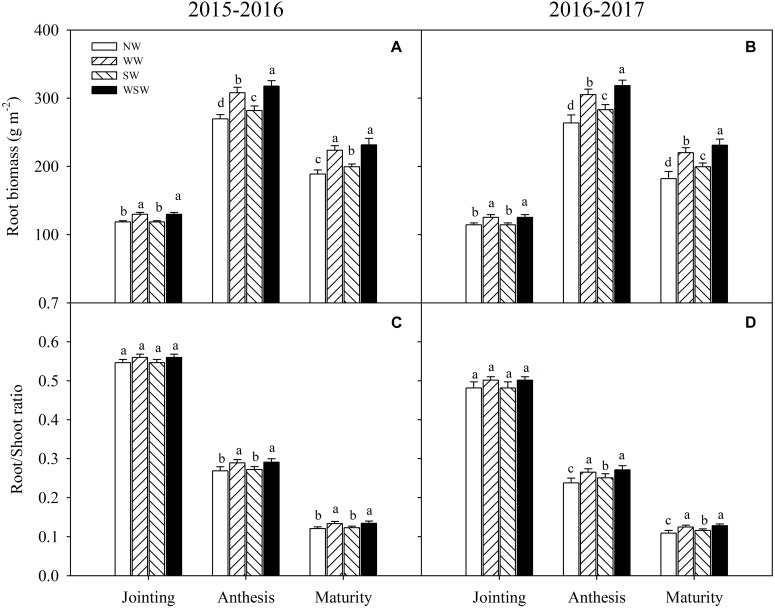
Root biomass **(A,B)** and root/shoot ratio **(C,D)** affected by winter and spring night-warming in 2015–2017. NW, WW, SW, and WSW refer to no warming control, winter night-warming, spring night-warming, and winter + spring night-warming, respectively. Lower case letters refer to significant difference between treatments (*P* < 0.05). Whiskers on the top of the bars indicate standard error.

At jointing stage, root biomass was significantly increased in WW and WSW compared with NW (Figure 3A,B). At anthesis and maturity stages, root biomass was significantly increased in night-warming treatments compared with NW, and WSW (increased 42.86–54.66 g m^−2^) resulted in higher increases than WW (increased 34.82–41.43 g m^−2^) and SW (increased 10.51–19.24 g m^−2^).

At jointing stage, root/shoot ratio was slightly increased in WW and WSW compared with NW, but the difference was not significant (Figure 3C,D). At anthesis and maturity stages, root/shoot ratio was significantly increased in night-warming treatments compared with NW, except SW in 2015–2016. The increases were higher in WSW than WW and SW.

### Root Dry Matter Accumulation Rate and Root Weight Density

**FIGURE 4:**
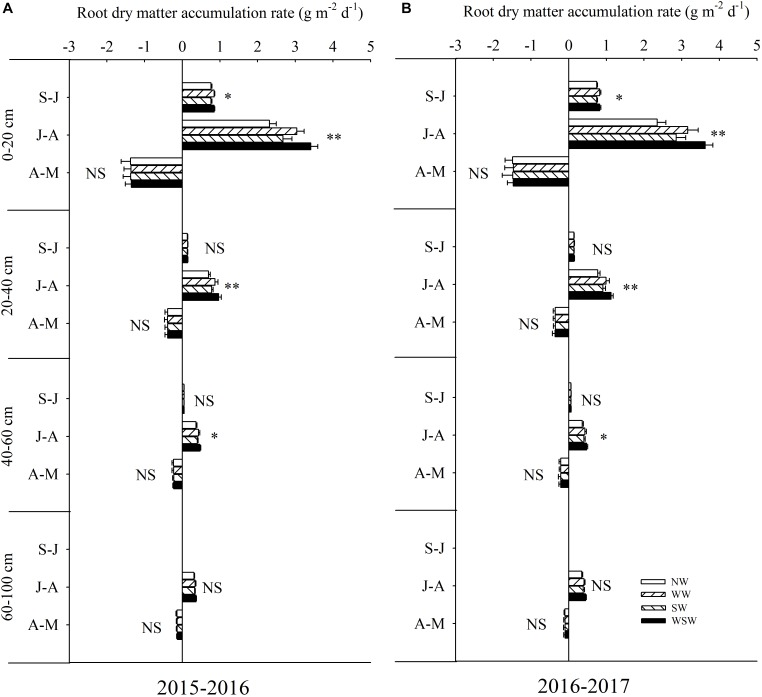
Root dry matter accumulation rate in different soil layers during different growth periods affected by winter and spring night-warming in 2015–2016 **(A)** and 2016–2017 **(B)**. NW, WW, SW, and WSW refer to no warming control, winter night-warming, spring night-warming, and winter + spring night-warming, respectively. S–J, J–A, and A–M refer to the growth period of sowing to jointing, jointing to anthesis and anthesis to maturity, respectively. ^∗∗^ and ^∗^ indicate significant difference between treatments and control at the 0.01 and 0.05 level and NS indicate no significant difference.

From sowing to jointing (S-J), root dry matter accumulation rate in the 0–20 cm soil layer was increased in WW and WSW compared with NW, while root dry matter accumulation rate in the 20–60 cm soil layer was not significant among all treatments (Figure 4). From jointing to anthesis (J-A), root dry matter accumulation rate in the 0–60 cm soil layer was increased in night-warming treatments compared with NW, and WSW (increased 0.10–1.28 g m^−2^ d^−1^) resulted in higher increases than WW (increased 0.06–0.81 g m^−2^ d^−1^) and SW (increased 0.03–0.36 g m^−2^ d^−1^). Root dry matter accumulation rate in the 60–100 cm soil layer was not significant among all treatments. Moreover, the increases of root dry matter accumulation rate in the 0–20 cm soil layer (increased 0.36–1.27 g m^−2^ d^−1^) were obviously higher than 20–40 cm soil layer (increased 0.09–0.35 g m^−2^ d^−1^) and 40–60 cm soil layer (increased 0.03–0.12 g m^−2^ d^−1^). From anthesis to maturity (A-M), root dry matter accumulation rate in the 0–100 cm soil layer was not significant among all treatments.

**FIGURE 5:**
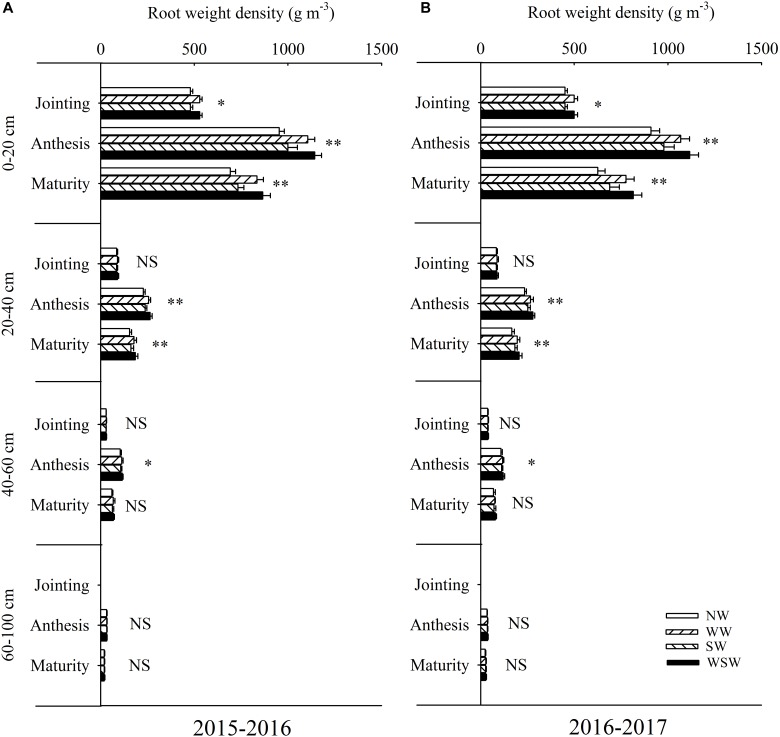
Root weight density in different soil layers at different growth stages affected by winter and spring night-warming in 2015–2016 **(A)** and 2016–2017 **(B)**. NW, WW, SW and WSW refer to no warming control, winter night-warming, spring night-warming and winter + spring night-warming, respectively. ^∗∗^ and ^∗^ indicate significant difference between treatments and control at the 0.01 and 0.05 level and NS indicate no significant difference. Whiskers on the top of the bars indicate standard error.

At jointing stage, root weight density in the 0–20 cm soil layer was increased in WW and WSW compared with NW, while root weight density in the 20–60 was not significant among all treatments (Figure 5). At anthesis stage, root weight density in the 0–60 cm soil layer was increased in night-warming treatments, and WSW (increased 11.22–207.09 g m^−3^) resulted in higher increases than WW (increased 8.33–159.59 g m^−3^) and SW (increased 2.86–46.94 g m^−3^). Root weight density in the 60–100 cm soil layer was not significant among all treatments. At maturity stage, root weight density in the 0–40 cm soil layer was increased in night-warming treatments, while root weight density in the 40–100 cm soil layer was not significant among all treatments.

### Plant N Uptake

From sowing to jointing (S-J), plant N uptake from basal ^15^N was increased in WW and WSW compared with NW (Table 2). From jointing to anthesis (J-A) and anthesis to maturity (A-M), plant N uptake from basal ^15^N was increased in night-warming treatments compared with NW, and WSW (increased 0.08–0.55 g m^−2^) resulted in higher increases than WW (increased 0.05–0.32 g m^−2^) and SW (increased 0.04–0.24 g m^−2^). Increases in the plant N uptake from basal ^15^N was higher from jointing to anthesis (increased 0.22–0.55 g m^−2^) than from sowing to jointing (increased 0.22–0.25 g m^−2^) and from anthesis to maturity (increased 0.04–0.14 g m^−2^). From jointing to anthesis (J-A) and anthesis to maturity (A-M), plant N uptake from top-dressed ^15^N was increased in night-warming treatments compared with NW, and WSW (increased 0.17–1.44 g m^−2^) resulted in higher increases than WW (increased 0.06–0.93 g m^−2^) and SW (increased 0.04–0.50 g m^−2^). Increases in the plant N uptake from top-dressed ^15^N was higher from jointing to anthesis (increased 0.45–1.44 g m^−2^) than from anthesis to maturity (increased 0.04–0.19 g m^−2^). Moreover, from sowing to maturity (S-M), increases in the plant N uptake from top-dressed ^15^N (increased 0.26–0.90 g m^−2^) was higher than from basal ^15^N (increased 0.53–1.61 g m^−2^).

**Table 2:** Plant N uptake from fertilizer ^15^N and soil N during different growth periods affected by winter and spring night-warming in 2015–2017.

Item	Treatment	S-J		J-A		A-M		S-M	
		2015–2016	2016–2017	2015–2016	2016–2017	2015–2016	2016–2017	2015–2016	2016–2017
Basal ^15^N (g m^−2^)	NW	2.14b	2.10b	1.24c	1.38c	0.12b	0.25b	3.50c	3.73d
	WW	2.36a	2.35a	1.56b	1.66b	0.22a	0.30ab	4.14a	4.30b
	SW	2.14b	2.10b	1.48b	1.60b	0.20ab	0.29ab	3.82b	3.99c
	WSW	2.36a	2.35a	1.79a	1.79a	0.26a	0.33a	4.40a	4.47a
Top–dressed ^15^N (g m^−2^)	NW	–	–	5.08d	5.20d	0.53b	0.62c	5.61d	5.82d
	WW	–	–	6.01b	6.00b	0.59b	0.78a	6.61b	6.78b
	SW	–	–	5.58c	5.65c	0.57b	0.70b	6.14c	6.35c
	WSW	–	–	6.52a	6.35a	0.70a	0.81a	7.22a	7.16a
Fertilizer ^15^N (g m^−2^)	NW	2.14b	2.10b	6.32d	6.58d	0.65b	0.87c	9.11d	9.55d
	WW	2.36a	2.35a	7.58b	7.65b	0.81ab	1.08ab	10.75b	11.08b
	SW	2.14b	2.10b	7.06c	7.25c	0.76b	0.99b	9.96c	10.34c
	WSW	2.36a	2.35a	8.31a	8.14a	0.95a	1.14a	11.62a	11.63a
Soil N (g m^−2^)	NW	4.63a	5.55a	2.35a	2.67a	1.42c	0.85b	8.40b	9.06b
	WW	4.77a	5.71a	2.31a	2.74a	1.55b	0.89b	8.62ab	9.34a
	SW	4.63a	5.55a	2.37a	2.77a	1.45bc	0.88b	8.45b	9.20ab
	WSW	4.77a	5.71a	2.36a	2.70a	1.70a	0.96a	8.83a	9.37a
Total uptake N (g m^−2^)	NW	6.77b	7.65b	8.66c	9.25c	2.07c	1.72c	17.50c	18.61d
	WW	7.13a	8.06a	9.88b	10.39b	2.36b	1.97ab	19.37b	20.42b
	SW	6.77b	7.65b	9.43b	10.02b	2.21bc	1.87b	18.41bc	19.54c
	WSW	7.13a	8.06a	10.68a	10.84a	2.65a	2.10a	20.45a	21.00a

*NW, WW, SW and WSW refer to no warming control, winter night-warming, spring night-warming, and winter + spring night-warming, respectively. S-J, J-A, A-M, and S-M refer to the growth period of sowing to jointing, jointing to anthesis, anthesis to maturity and sowing to maturity, respectively. Lower case letters refer to significant difference between treatments (P < 0.05).*

Plant N uptake from fertilizer ^15^N was the sum of plant N uptake from basal ^15^N and top-dressed ^15^N. Similarly, plant N uptake from fertilizer ^15^N during each growth stage was also increased in night-warming treatments compared with NW, especially during jointing to anthesis (J-A), and WSW resulted in higher increases than WW and SW. Throughout the growing season, plant N uptake from soil N during anthesis to maturity (A-M) was increased in night-warming treatments compared with NW, while plant N uptake from soil N during sowing to jointing (S-J) and jointing to anthesis (J-A) was not significant among all treatments. Total plant N uptake was the sum of plant N uptake from fertilizer ^15^N and soil N. Total plant N uptake during each growth stage was increased in night-warming treatments compared with NW, especially during jointing to anthesis (J-A), and WSW resulted in higher increases than WW and SW.

### Residual Fertilizer ^15^N Content

**FIGURE 6:**
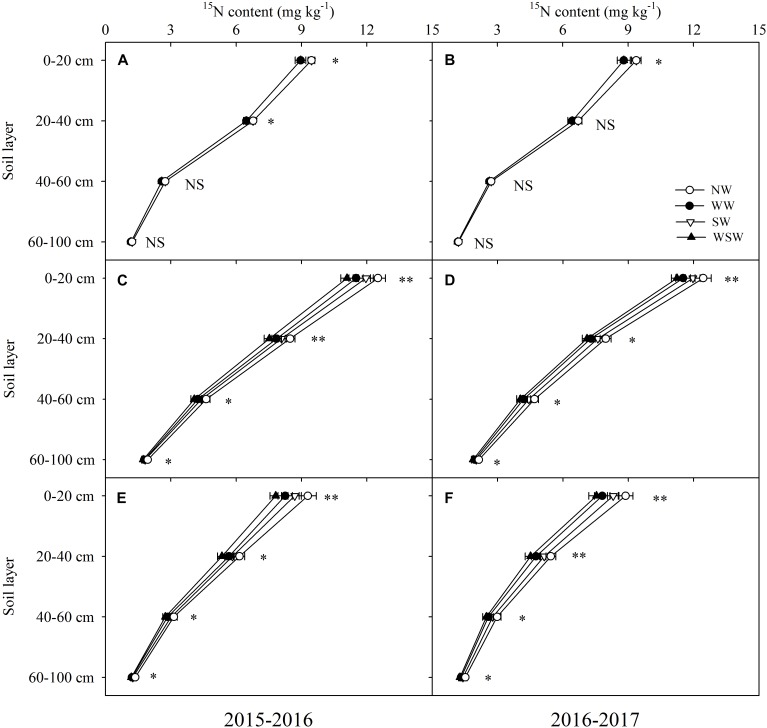
Residual fertilizer ^15^N content in different soil layers at jointing **(A,B)**, anthesis **(C,D)**, and maturity **(E,F)** affected by winter and spring night-warming in 2015–2017. NW, WW, SW, and WSW refer to no warming control, winter night-warming, spring night-warming, and winter + spring night-warming, respectively. ^∗∗^ and ^∗^ indicate significant difference between treatments and control at the 0.01 and 0.05 level, and NS indicate no significant difference. Whiskers on the top of the bars indicate standard error.

At jointing stage, residual fertilizer ^15^N content in the 0–40 cm soil layer was decreased in WW and WSW compared with NW, except in the 20–40 cm soil layer in 2016–2017 (Figure 6A,B). At anthesis and maturity stages, residual fertilizer ^15^N content in the 0–100 cm soil layer was decreased in night-warming treatments compared with NW (Figure 6C–F), and WSW (decreased 0.18–1.47 mg kg^−1^) resulted in higher decreases than WW (decreased 0.15–1.07 mg kg^−1^) and SW (decreased 0.11–0.60 mg kg^−1^). Moreover, decreases in residual fertilizer ^15^N content in the 0–20 cm (decreased 0.48–1.47 mg kg^−1^) soil layer was higher than in the 20–40 cm (decreased 0.31–0.95 mg kg^−1^), 40–60 cm (decreased 0.13–0.65 mg kg^−1^) and 60–100 cm (decreased 0.11–0.26 mg kg^−1^) soil layers.

### Losses of ^15^N Fertilizer

From sowing to jointing (S-J) and anthesis to maturity (A-M), the losses of basal ^15^N were slightly decreased in WW and WSW compared with NW, but the difference was not significant (Table 3). From jointing to anthesis (J-A), the losses of basal ^15^N were decreased in night-warming treatments compared with NW, and WSW (decreased 0.22–0.38 g m^−2^) resulted in higher decreases than WW (decreased 0.16–0.22 g m^−2^) and SW (decreased 0.08–0.11 g m^−2^). From jointing to anthesis (J-A) and anthesis to maturity (A-M), the losses of top-dressed ^15^N were decreased in night-warming treatments compared with NW, and WSW (decreased 0.22–0.77 g m^−2^) resulted in higher decreases than WW (decreased 0.15–0.49 g m^−2^) and SW (decreased 0.10–0.21 g m^−2^). Decreases in the losses of top-dressed ^15^N were higher from jointing to anthesis (decreased 0.19–0.77 g m^−2^) than from anthesis to maturity (decreased 0.10–0.33 g m^−2^). Moreover, from sowing to maturity (S-M), decreases in the losses of top-dressed ^15^N (decreased 0.29–1.11 g m^−2^) was higher than basal ^15^N (decreased 0.10–0.52 g m^−2^).

**Table 3:** ^15^N losses of basal and top-dressed fertilization during different growth periods affected by winter and spring night-warming in 2015–2017.

Item	Treatment	S-J		J-A		A-M		S-M	
		2015–2016	2016–2017	2015–2016	2016–2017	2015–2016	2016–2017	2015–2016	2016–2017
Basal ^15^N (g m^−2^)	NW	3.53a	3.63a	1.73a	1.45a	0.86a	0.80a	6.12a	5.88a
	WW	3.46a	3.54a	1.51bc	1.29bc	0.81a	0.76a	5.77ab	5.59b
	SW	3.53a	3.63a	1.62ab	1.37ab	0.81a	0.78a	5.96ab	5.78ab
	WSW	3.46a	3.54a	1.35c	1.23c	0.79a	0.75a	5.60b	5.53b
Top–dressed ^15^N (g m^−2^)	NW	–	–	1.54a	1.53a	0.92a	1.02a	2.47a	2.54a
	WW	–	–	1.05bc	1.20bc	0.74b	0.87bc	1.79bc	2.06bc
	SW	–	–	1.33ab	1.34ab	0.82ab	0.91b	2.14ab	2.25b
	WSW	–	–	0.77c	0.99c	0.59c	0.80c	1.36c	1.79c
Fertilizer ^15^N (g m^−2^)	NW	3.53a	3.63a	3.27a	2.97a	1.78a	1.82a	8.58a	8.42a
	WW	3.46a	3.54a	2.56bc	2.48bc	1.56bc	1.62b	7.57bc	7.65bc
	SW	3.53a	3.63a	2.95ab	2.70ab	1.63ab	1.69ab	8.10ab	8.03ab
	WSW	3.46a	3.54a	2.12c	2.22c	1.38c	1.55b	6.96c	7.32c

*NW, WW, SW, and WSW refer to no warming control, winter night-warming, spring night-warming and winter + spring night-warming, respectively. S-J, J-A, A-M, and S-M refer to the growth period of sowing to jointing, jointing to anthesis, anthesis to maturity and sowing to maturity, respectively. Lower case letters refer to significant difference between treatments (P < 0.05).*

From sowing to jointing (S-J), the losses of fertilizer ^15^N were slightly decreased in WW and WSW compared with NW, but the difference was not significant. From jointing to anthesis (J-A) and anthesis to maturity (A-M), the losses of fertilizer ^15^N were decreased in night-warming treatments compared with NW, and WSW resulted in higher decreases than WW and SW.

### Fates of Basal and Top-Dressed ^15^N

**FIGURE 7:**
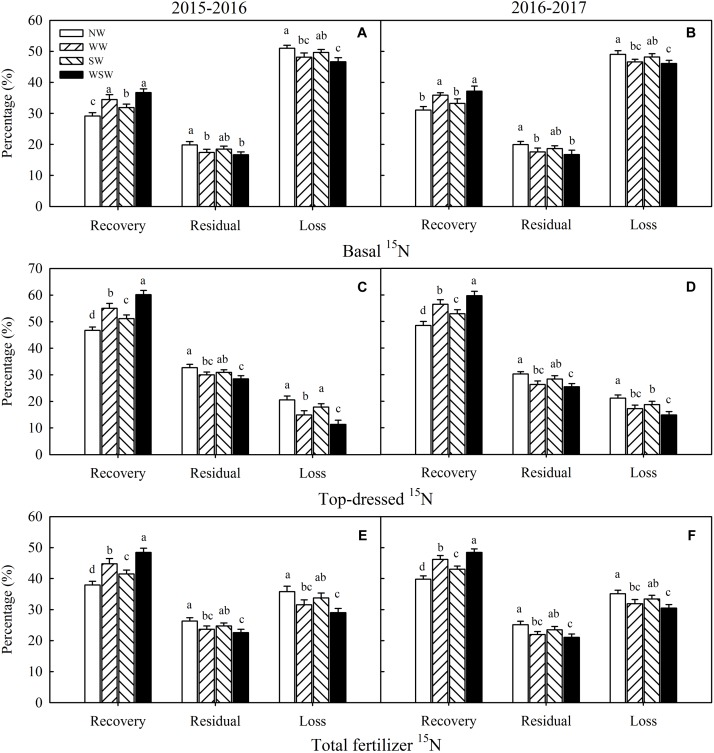
The fates of basal ^15^N **(A,B)**, top–dressed ^15^N **(C,D)**, and fertilizer ^15^N **(E,F)** at maturity affected by winter and spring night-warming in 2015–2017. NW, WW, SW, and WSW refer to no warming control, winter night-warming, spring night-warming, and winter + spring night-warming, respectively. Lower case letters refer to significant difference between treatments (*P* < 0.05). Whiskers on the top of the bars indicate standard error.

The recovery of basal ^15^N was increased in night-warming treatments compared with NW (Figure 7A,B), and WSW (increased 6.13–7.55%) resulted in higher increases than WW (increased 4.78–5.34%) and SW (increased 2.69–3.56%). On the contrary, the residual and loss of basal ^15^N were decreased in night-warming treatments compared with NW, and WSW resulted in higher decreases than WW and SW. The recovery of top-dressed ^15^N was increased in night-warming treatments compared with NW (Figure 7C,D), and WSW (increased 11.18–13.39%) resulted in higher increases than WW (increased 7.95–8.31%) and SW (increased 4.40–4.44%). The residual and loss of top-dressed ^15^N were decreased in night-warming treatments compared with NW, and WSW resulted in higher decreases than WW and SW. Moreover, the recovery increases of top-dressed ^15^N (increased 4.40–13.39%) were higher than those of basal ^15^N (increased 2.13–7.55%), and the residual and loss decreases of top-dressed ^15^N (decreased 1.74–9.19%) were also higher than those of basal ^15^N (decreased 0.84–4.34%). Similarly, the recovery of fertilizer ^15^N was increased in night-warming treatments compared with NW, while the residual and loss of fertilizer ^15^N were decreased (Figure 7E,F).

### Path Analysis Between Root Dry Matter Distribution and Losses of Total Fertilizer ^15^N

The path analysis was conducted to quantify the roles of root dry matter distribution in different soil layers in contribution to losses of total fertilizer ^15^N. At jointing and anthesis stages, the direct path coefficient of root dry matter distribution in 0–20 cm (0.6325–0.6391) soil layer was larger than 20–40 cm (0.1941–0.4341) and 40–60 cm (0.1561–0.3658) soil layers, indicating that root dry matter distribution in 0–20 cm soil layer was the most important in contributing to reducing losses of total fertilizer ^15^N (Table 4). Moreover, at jointing stage, the direct path coefficient of root dry matter distribution in 0–20 cm (0.6325) soil layer was little larger than in 20–40 cm (0.4341) and 40–60 cm (0.3658) soil layers, while at anthesis stage, the direct path coefficient of root dry matter distribution in 0–20 cm (0.6391) soil layer was highly larger than in 20–40 cm (0.1941) and 40–60 cm (0.1651) soil layers, indicating that anthesis stage was more important than jointing stage in contribution to reducing losses of total fertilizer ^15^N. The path coefficient of root dry matter distribution in 60–100 cm (0.0241) soil layer was obviously lower than other soil layers, indicating that root dry matter distribution in 60–100 cm soil layer had marginal effects in contribution to reducing losses of total fertilizer ^15^N.

**Table 4:** Path analysis between root dry matter distribution in different soil layers and losses of total fertilizer ^15^N (from sowing to maturity) affected by winter and spring night-warming in 2015–2017.

	Jointing				Anthesis			
Soil layer (cm)	0–20 →^15^N losses	20–40 →^15^N losses	40–60 →^15^N losses	60–100 →^15^N losses	0–20 →^15^N losses	20–40 →^15^N losses	40–60 →^15^N losses	60–100 →^15^N losses
0–20	**-0.6325**	−0.3213	0.1502	–	**−0.6391**	−0.1652	−0.1224	0.0033
20–40	**−0.4681**	−0.4341	0.1524	–	**−0.5440**	−0.1941	−0.1502	0.0107
40–60	0.2596	0.1808	**−0.3658**	–	**−0.4738**	−0.1766	−0.1651	0.0092
60–100	–	–	–	–	**−0.0870**	−0.0863	−0.0631	0.0241

*The data with an underline represent direct path coefficients, and those in bold represent the most important path coefficients that determine ^15^N losses.*

### Grain Yield

**FIGURE 8:**
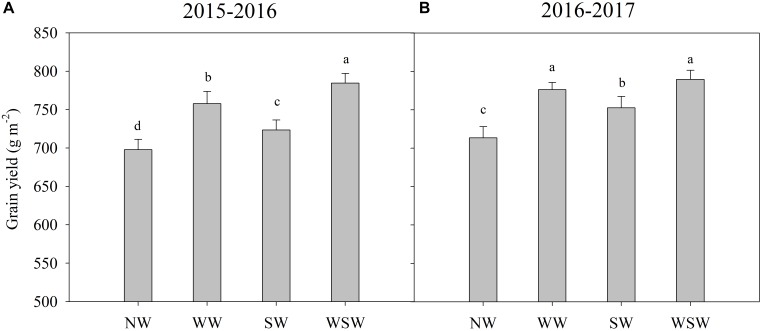
Grain yield affected by winter and spring night-warming in 2015–2016 **(A)** and 2016–2017 **(B)**. NW, WW, SW, and WSW refer to no warming control, winter night-warming, spring night-warming and winter + spring night-warming, respectively. Lower case letters refer to significant difference between treatments (*P* < 0.05). Whiskers on the top of the bars indicate standard error.

Grain yields were 697.96, 757.76, 723.47, and 784.49 g m^−2^ for treatments NW, WW, SW, and WSW in 2015–2016 and were 713.61, 776.46, 752.55, and 789.46 g m^−2^ for treatments NW, WW, SW, and WSW in 2016–2017 (Figure 8). The grain yield of winter wheat was increased in night-warming treatments compared with NW, and WSW resulted in higher increases than WW and SW. The grain yield of WW, SW, and WSW were 8.57–8.81%, 3.65–5.46%, and 10.62–12.39% higher than NW.

## Discussion

In the present study, winter and spring night-warming reduced the losses of total fertilizer ^15^N and improved the recovery of total fertilizer ^15^N, while maximizing the grain yield of winter wheat (Figure 7, 8). Moreover, winter + spring night-warming (increased 10.62–12.39%) resulted in higher increases of grain yield than winter night-warming (increased 8.57–8.81%) and spring night-warming (increased 3.65–5.46%), which was consistent with our previous studies (Fan et al., 2015; Hu et al., 2018).

Using labeled ^15^N-fertilizer can directly quantify the fates of N fertilizer (Shu et al., 2012; Ruisi et al., 2016). This method has been widely used to study the N fertilizer use efficiency (Harmsen, 2003). Some studies have shown that plant N uptake could be affected by climate warming (Nam et al., 2013; Zhang et al., 2013; Xu et al., 2015), while the fates of ^15^N fertilizer (recovery, residual, and loss) in response to asymmetric warming is still not clear. In the present study, plant N uptake from basal ^15^N and top-dressed ^15^N was increased in night-warming treatments compared with NW during each growth stage, and WSW resulted in higher increases than WW and SW (Table 2). Throughout the growing season, increases in the plant N uptake from basal ^15^N and top-dressed ^15^N were higher from jointing to anthesis (S-J) than from sowing to jointing (S-J) and from anthesis to maturity (A-M), which was associated with highly improved root dry matter accumulation rate during this stage (Table 2 and Figure 4). Moreover, from sowing to maturity (S-M), increases in the plant N uptake from top-dressed ^15^N (increased 0.26–0.90 g m^−2^) was higher than from basal ^15^N (increased 0.53–1.61 g m^−2^), indicating that night-warming treatments improved plant N uptake from fertilizer ^15^N mainly by top-dressed ^15^N during jointing to anthesis (Table 2). Furthermore, the main stage of plant N uptake from basal ^15^N is sowing to jointing, while the main stage of plant N uptake from top-dressed ^15^N is jointing to anthesis. The plant N uptake from soil N was only increased during anthesis to maturity (A-M) in night-warming treatments compared with NW, which may be due to the large amount of fertilizer ^15^N uptake before anthesis (Table 2). The increased plant N uptake of fertilizer ^15^N helped improve recovery of fertilizer ^15^N and reduce losses of fertilizer ^15^N.

The residual fertilizer ^15^N content in the soil layer can reflect the movement of N fertilizer (Wu et al., 2009). In the present study, at anthesis and maturity stages, residual fertilizer ^15^N content in the 0–100 cm soil layer was decreased in night-warming treatments compared with NW (Figure 6C–F), and WSW (decreased 0.18–1.47 mg kg^−1^) resulted in higher decreases than WW (decreased 0.15–1.07 mg kg^−1^) and SW (decreased 0.11–0.60 mg kg^−1^). The decreases in residual fertilizer ^15^N content in the 0–20 cm (decreased 0.48–1.47 mg kg^−1^) soil layer was higher than in the 20–40 cm (decreased 0.31–0.95 mg kg^−1^), 40–60 cm (decreased 0.13–0.65 mg kg^−1^) and 60–100 cm (decreased 0.11–0.26 mg kg^−1^) soil layers, which was associated with highly improved root weight density in this soil layer (Figure 5, 6). Moreover, residual fertilizer^15^N content was not significant in 40–100 cm soil layer among all treatments at jointing stage (Figure 6A,B), while residual fertilizer ^15^N content was reduced in night-warming treatments at anthesis and maturity stages (Figure 6C–F), indicating that improved root weight density prevents the downward movement of fertilizer ^15^N, which favored reducing leaching losses of fertilizer ^15^N. Furthermore, the residual fertilizer^15^N content in the 0–20 cm (7.54–12.51 mg kg^−1^) soil layer was higher than in the 20–40 cm (4.51–8.48 mg kg^−1^), 40–60 cm (2.48–4.70 mg kg^−1^) and 60–100 cm (1.19–2.14 mg kg^−1^) soil layers, indicating that most of the fertilizer ^15^N remained in the 0–20 cm soil layer (Figure 6).

Many studies have reported that high N fertilizer losses resulted in serious environmental impacts, such as eutrophication of surface waters, nitrate pollution of groundwater and greenhouse gas emissions (Ju et al., 2009; Townsend and Howarth, 2010). In the present study, the losses of basal ^15^N and top-dressed ^15^N from sowing to maturity (S-M) were decreased in night-warming treatments compared with NW, and WSW (decreased 0.35–1.11 g m^−2^) resulted in higher decreases than WW (decreased 0.29–0.68 g m^−2^) and SW (decreased 0.10–0.33 g m^−2^) (Table 3). The decreases in the losses of top-dressed ^15^N (decreased 0.29–1.11 g m^−2^) was higher than basal ^15^N (decreased 0.10–0.52 g m^−2^), indicating that night-warming treatments decreased losses of fertilizer ^15^N mainly by decreasing losses of top–dressed ^15^N. Moreover, decreases of top-dressed ^15^N losses from jointing to anthesis (decreased 0.19–0.77 g m^−2^) were higher than from anthesis to maturity (decreased 0.10–0.33 g m^−2^), which was associated with improved root dry matter accumulation rate during this period (Table 3 and Figure 4). Furthermore, the loss of top-dressed ^15^N (46.07–50.97%) was higher than basal ^15^N (11.36–21.19%), indicating that the loss of fertilizer ^15^N mainly came from basal ^15^N (Figure 7). The further analysis showed that the losses of basal ^15^N from sowing to jointing accounted for most of the losses of fertilizer ^15^N (more than 40%) in two growing seasons (Table 3), which was due to root grew slowly at the early growth stage (Wang et al., 2014).

A good root system could improve plant N uptake of wheat and reduce N losses (Palta et al., 2007; Foulkes et al., 2009). It has been reported that root growth could be strongly affected by climate warming (Bai et al., 2010). In the present study, root dry matter accumulation rate was increased in night-warming treatments, resulted in increased root biomass at jointing, anthesis and maturity stages, which helped increase recovery of total fertilizer ^15^N and reduce losses of total fertilizer ^15^N (Figure 3, 4). Moreover, the root biomass had a better response than shoot biomass under night-warming, resulted in increased root/shoot ratio at anthesis and maturity stages (Figure 3). Hou et al. (2018) reported that warming significantly increased root biomass under two tillage systems, and in till system root biomass was increased in the deeper soil layers, while in no-till system root biomass was increased in the surface layer. In the present study, the increases of root dry matter accumulation rate in the 0–20 cm soil layer (increased 0.36–1.27 g m^−2^ d^−1^) were obviously higher than 20–40 cm soil layer (increased 0.09–0.35 g m^−2^ d^−1^) and 40–60 cm soil layer (increased 0.03–0.12 g m^−2^ d^−1^), indicating that the increased root biomass was mainly caused by improved pre-anthesis root dry matter accumulation rate in the 0–20 cm soil layer (Figure 4). Moreover, root weight density was highly improved in this soil layer, which favored reducing leaching losses of total fertilizer ^15^N (Figure 5). Furthermore, in the present study, root dry matter distribution in 0–20 cm soil layer was the most important in contributing to reducing losses of total fertilizer ^15^N, and root dry matter accumulation in 0–20 cm soil layer at anthesis was more important than at jointing stage in contributing to reducing losses of total fertilizer ^15^N (Table 4), indicating that the reduced losses of total fertilizer ^15^N was mainly caused by highly improved root dry matter distribution in 0–20 cm soil layer from jointing to anthesis (J-A). Therefore, optimization of N fertilizer management, for example, decreasing basal/top-dressing ratio of N fertilizer properly, to regulate the root growth and distribution during jointing to anthesis, a time when wheat roots acquire most top-dressed ^15^N, may be a good strategy for high wheat NUE and minimizing the environmental impact of N losses under future warming condition.

## Conclusion

Winter and spring night-warming reduced the losses of total fertilizer ^15^N, which was mainly caused by reduced losses of top-dressed ^15^N during jointing to anthesis, and WSW resulted in higher advantages than WW and SW. Winter and spring night-warming promoted pre-anthesis root growth in 0–60 cm soil layer, resulted in increased recovery of fertilizer ^15^N and reduced losses of fertilizer ^15^N. Moreover, root distribution in 0–60 cm soil layer was improved in response to night-warming, resulted in reduced leaching losses of fertilizer ^15^N. Furthermore, root distribution in 0–20 cm soil layer was the most important in contributing to reducing losses of total fertilizer ^15^N compared with other soil layers. The findings of this study should be considered to develop efficient N management strategies for high wheat NUE and minimizing the environmental impact of N losses under future climate change.

## Author Contributions

ZT and TD designed the experiments. CH and ZT conducted the study, collected and analyzed the data, and prepared the draft. SS, JY, YY, and HG helped in sampling and the measurements of the parameters. DJ and WC helped in drafting the manuscript and the interpretation of the results.

## Conflict of Interest Statement

The authors declare that the research was conducted in the absence of any commercial or financial relationships that could be construed as a potential conflict of interest.

## Abbreviations

N: nitrogen
NUE: N use efficiency.
